# A Prediction Model of Defecation Based on BP Neural Network and Bowel Sound Signal Features

**DOI:** 10.3390/s22187084

**Published:** 2022-09-19

**Authors:** Tie Zhang, Zequan Huang, Yanbiao Zou, Jun Zhao, Yuwei Ke

**Affiliations:** 1 School of Mechanical and Automotive Engineering, South China University of Technology, Guangzhou 510641, China; 2 China Rehabilitation Research Center, Beijing 100000, China

**Keywords:** bowel sounds, defecation prediction, signal processing, feature extraction, BP classifier

## Abstract

(1) Background: Incontinence and its complications pose great difficulties in the care of the disabled. Currently, invasive incontinence monitoring methods are too invasive, expensive, and bulky to be widely used. Compared with previous methods, bowel sound monitoring is the most commonly used non-invasive monitoring method for intestinal diseases and may even provide clinical support for doctors. (2) Methods: This paper proposes a method based on the features of bowel sound signals, which uses a BP classification neural network to predict bowel defecation and realizes a non-invasive collection of physiological signals. Firstly, according to the physiological function of human defecation, bowel sound signals were selected for monitoring and analysis before defecation, and a portable non-invasive bowel sound collection system was built. Then, the detector algorithm based on iterative kurtosis and the signal processing method based on Kalman filter was used to process the signal to remove the aliasing noise in the bowel sound signal, and feature extraction was carried out in the time domain, frequency domain, and time–frequency domain. Finally, BP neural network was selected to build a classification training method for the features of bowel sound signals. (3) Results: Experimental results based on real data sets show that the proposed method can converge to a stable state and achieve a prediction accuracy of 88.71% in 232 records, which is better than other classification methods. (4) Conclusions: The result indicates that the proposed method could provide a high-precision defecation prediction result for patients with fecal incontinence, so as to prepare for defecation in advance.

## 1. Introduction

Incontinence is a common geriatric syndrome. Incontinence and its complications not only bring pain to the disabled elderly but also increase the risk of stress skin injury and seriously affect the quality of life of the elderly [1]. From a medical perspective, incontinence can be caused by a variety of physiological factors, and any factor can be affected by motor and cognitive dysfunction. Little is known about the exact mechanism behind incontinence, and the public is often accompanied by wrong subjective judgments about incontinence in clinical work, which leads to a generally low detection rate of incontinence in the elderly [2].

Effective identification and prediction of fecal incontinence is helpful for patients to choose the best treatment method, giving patients the possibility of a successful cure. However, few physiological signals have been used to predict fecal incontinence [3]. The key to predicting incontinence is that it is usually the result of undetected defecation in the rectum, rather than due to muscle or nerve defects [4]. In general treatment studies, anorectal measurement has been found to provide suspected diagnostic information or evidence for patients with incontinence [5], as anorectal manometry provides comprehensive information about the anorectal function and evaluate anorectal sensory response, rectal inhibitory reflex, and rectal compliance. Therefore, some doctors obtain data on rectal muscle force in the form of rectal palpation by placing their hands into the anus [6]. In 2008, the American Society of Neurogastroenterology and Gastrointestinal Dynamics took the contraction waves generated by intestinal peristalsis as the main basis for the generation of defecation [7,8]. Aiming at the reconstruction of rectal sensory function and biomechanical compatibility, Mantoo et al. [9] proposed an electromagnetic artificial anal sphincter in 2012, and Zhang [10] realized the reconstruction of rectal perception function; the simulation results show that artificial anal sphincters can achieve defecation prediction with high accuracy. In 2013, Jelovsek also used a predictive model to assess the risk of postpartum urinary and fecal incontinence [11]. Although the ability to subclassify mechanisms of incontinence has improved in recent years, most advanced equipment requires highly skilled operators to implement and interpret the results, which limits feasibility and translation into routine practice.

More importantly, the current fecal incontinence prediction method based on anorectal function may not only cause secondary injury to incontinence patients but also affect their physical function due to its invasiveness. In order to alleviate the disgust and embarrassment of patients with fecal incontinence, based on existing research in these fields, a further hypothesis about the prediction of fecal incontinence can be generated: The search for a pre-prediction method of non-invasive incontinence for the care of elders is expected to become a possible new research direction of this field.

At the same time, bowel sound (BS) signals are proposed as an alternative medical research method to overcome the obstacles mentioned above. BS signal is clinically used as a useful indicator of intestinal peristalsis. It is the most common method to detect and monitor intestinal diseases. It can be used to diagnose acute intestinal obstruction, renal failure [12], and acute appendicitis—all of which can lead to acute reduced intestinal peristalsis in the abdomen. In some recent studies, Yoshino et al. [13] used a single channel filter to collect intestinal sounds in 21 patients with mechanical intestinal obstruction, proving that the monitoring of intestinal sounds characteristics may provide an objective basis for the evaluation of the severity of intestinal obstruction. Dalle et al. [14] recorded the bowel sounds of 67 subjects aged 17–88 years and analyzed the bowel sounds with the method of adjustable grid, finding that the severity and progression of intestinal obstruction could be more accurately determined by combining the patients’ bowel sounds, so as to help clinicians make corresponding medical decisions. Kim et al. [15] used artificial neural network analysis to take patients with constipation as the research object to compare the analysis results obtained by bowel sound detector with colon transmission tests. The results show that there was a good correlation between bowel sound algorithm and colon transmission tests.

Therefore, we propose the following hypothesis: Using an appropriate machine learning algorithm, such as the BP neural network, we can classify incontinent people through bowel sound information. In fact, the BP neural network is one of the most commonly used classification algorithms and shows better performance than other machine learning methods in solving linear classification. In previous studies, machine learning has been widely used in classification fields and achieved good diagnostic results. In 2004, Fernandez [16] pointed out that there has been a lot of work to design good classifiers from different angles, and these works have achieved good results in many fields. In 2022, AL-Ghamdi [17] developed an Automated Outlier Detection for CyberSecurity in Higher Education Institutions (AOD-CSHEI) technique in order to determine the presence of intrusions or attacks in higher security institutions. In 2008, Garcia-Pedrajas [18] proposed a model applied using a neural network, the results are favorable. In 2013, Joseph [19] used a decision tree to analyze the clinical prediction rules of bowel sounds used to produce pathological enterocele. In this algorithm, the successful prediction rate was 93.2%.

In actual measurements, the bowel sound signals obtained by auscultation recorder can be assumed to be consist of a combination of bowel sound signal (BS) and background signal (BGS). BSs generated by intestinal movement are highly related to intestinal dysfunction. However, due to the non-stationary characteristics of these signals and their continuous changes in time and amplitude, weak level, and narrow band, it is difficult to extract these signals from strong EMG noise and environmental interference, and it is even more difficult to process the signals. Some scholars use adaptive filtering, wavelet transform, detectors based on fractal dimensions, and autoregressive and neuro-fuzzy modeling to detect BSs [20,21,22,23,24,25], including autoregressive modeling based on high-order statistics, wavelet transform, and neuro-fuzzy modeling. Rekanos [26] and others use the detection algorithm based on iterative kurtosis with stronger denoising performance, which greatly improved the reliability of the bowel sound detector. Sandler [27] used signal envelope and amplitude threshold processing to eliminate noise in BS recording with good results. However, the problem of the aliasing of bowel sound signal and noise is still not solved.

To sum up, the main challenge is to extract interesting features from measure signals with random noise and interference. The contributions of this paper are:

- **This paper proposes a prediction model of defecation based on the bowel sounds signal features and BP neural netwok**;
  This paper proposes a prediction model of defecation based on the bowel sounds signal features and BP neural network. Compared with the traditional machine learning methods, the biggest contribution of this paper is the combination of BP neural network with a bowel sound signal processing method, including iterative kurtosis and Kalman filter. In addition, other contributions in this paper include calculating the time domain, frequency domain, and time–frequency-domain features of the measured bowel sound signals and designing a neural network classification model based on the bowel sound signal features.
- **This paper designs a non-invasive collection system based on bowel sound signals for real-time collection**;
  We collected bowel sound data from patients in Beijing Bo’ai Hospital affiliated with the China Rehabilitation Research Center to ensure the validity of data.

**Figure 1:**
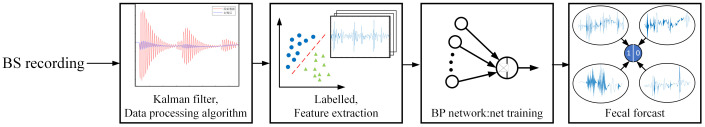
Research framework of this paper.

Although bowel sound auscultation is recognized as an important component of proper physical examination, its clinical value remains largely unstudied and subjectivized. The purpose of this study is to determine the reliability of the defecation prediction model trained on bowel sound features. The specific steps are shown in Figure 1, as follows:

a. **Bowel sound signal processing method based on iterative kurtosis detector algorithm and Kalman filter**;
  The Kalman filter method is added to the traditional detector algorithm based on the iteration kurtosis of bowel sound signals, which is used to extract signals from strong EMG noise and environmental interference, distinguishing the bowel sound signal and the noise signal and improving the denoising performance of bowel sound signals.
b. **Feature extraction of bowel sound signals in the time domain, frequency domain, and time–frequency domain**,
  Including time-domain feature extraction, frequency domain feature extraction, and time–frequency domain feature extraction. By compressing time series, dimension reduction is realized to improve the efficiency of data mining. Secondly, feature extraction of time series has the function of denoising, while retaining the main features of time series, which can better reflect the changes of time series and improve the quality of data mining.
c. **Prediction model of defecation based on bowel sound features**.
  Combined with the BP neural network, a defecation prediction model was designed based on the features of bowel sound signal in the time domain, frequency domain, and time–frequency domain. The rest of this paper is arranged as follows: The second section introduces the non-invasive collection system based on bowel sound signal. The third section introduces the detector algorithm based on iterative kurtosis and the bowel sound signal processing method of the Kalman filter, which are used to extract the bowel sound signal, so as to improve the denoising performance of BS. The fourth section introduces the feature extraction method based on transformation and calculates the time-domain features, frequency–domain features and time–frequency–domain features of the measured bowel sound signal, which are used to reduce the dimension of the bowel sound signal and retain the features of the original signal. The fifth section introduces the structure and propagation process of the neural network and proposes a defecation prediction algorithm based on BS features. The sixth section introduces the analysis of experimental results and verifies the feasibility of the neural network.

## 2. Overview of the Bowel Sound Collection System

### 2.1. Bowel Sound Collection System

In one study, Radnitz [28] and Garner [29] found that the combination of BS signals with an electronic stethoscope has considerable therapeutic potential for irritable bowel syndrome.

**Figure 2:**
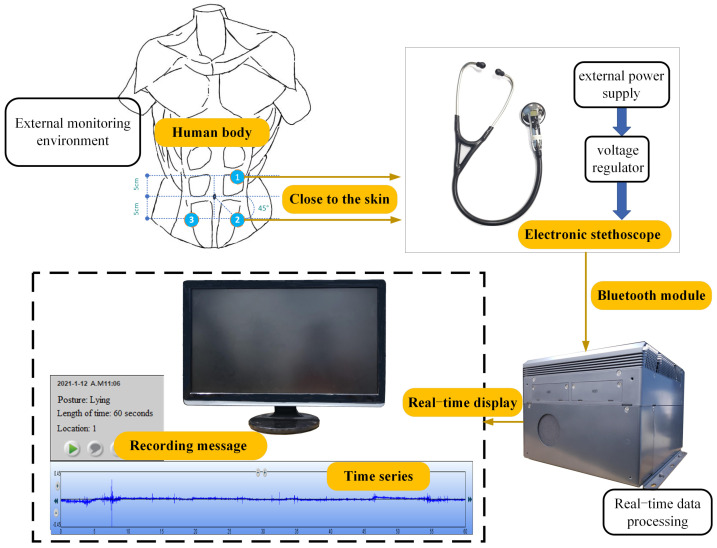
Hardware composition of the bowel sound collection system.

Figure 2 shows the structure of a bowel sound collection system. When collecting bowel sounds, the auscultation head was pressed against the skin, and the bowel sounds were collected from the ascending colon (position 1, 45° to the upper right of the navel), descending colon (position 2, 45° to the lower right of the navel), and sigmoid colon (position 3, 45° to the lower left of the navel) of the subjects’ abdomen.

The system includes the external monitoring environment, the signal collection module of the electronic stethoscope, and the real-time data processing module. The signal collection module of the electronic stethoscope collects the bowel sound signal from the external monitoring environment through the sensing and execution module and collects and transmits signals through the Bluetooth module. The real-time data processing module receives the signal sent by the signal collection module of the electronic stethoscope to process the signal, displaying the collected signal in real time through the upper computer, and sends out an alarm signal to remind the patient that the patient is currently in the pre-defecation state when necessary.

The sensing and execution module is placed close to the skin outside the body, replacing the traditional invasive treatment methods, such as artificial anal sphincter and intestinal pressure sensor. This non-invasive measurement mode makes the pre-defecation signal measurement and early warning have strong practical significance. Only this way can eliminate the pain of wound infection or surgical battery replacement caused by wire connection.

An electronic stethoscope is used to obtain the bowel sound signal of the patient, including a sensing module, an external power supply, and a voltage regulator, which ensure the normal operation of the sensing module. The purpose of the sensor execution module, whose bandwidth is 4000 Hz, is to selectively record the inherent bowel sound signal and minimize the bowel sound signal. The Bluetooth module is responsible for the signal collection and transmission. The electronic stethoscope is used for the bowel sound signal collection and the sensor signal conditioning.

### 2.2. Bowel Sound Signal Data Collection

The formation of the defecation signal depends on whether a large number of baroreceptors near the lower part of the rectal ampulla form a biological defecation signal under the comprehensive pressure stimulation generated by feces in this section of rectum. The key to the defecation prediction also lies in the comprehensive analysis of these pressure signals in different positions and directions, and the judgment of generation time of biological defecation signal [30].

**Figure 3:**
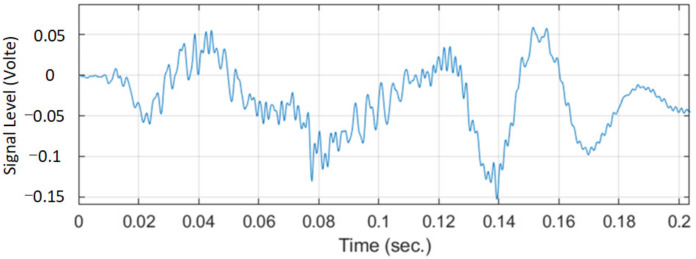
A bowel sound signal.

Figure 3 shows the bowel sound signal with a period of 0.2 s collected by electronic stethoscope. Collected signals are divided into normal regular breath sounds and irregular mutant intestinal sounds. The third section introduces the detector algorithm based on iterative kurtosis and the bowel sound signal processing method of Kalman filter to extract the bowel sound signal, so as to improve the denoising performance of BS.

## 3. Signal Processing Method Based on Bowel Sound Signal

In this section, we propose an iterative kurtosis-based detector algorithm and Kalman filtering method for extracting the mixed BS signal and noise signal at the same frequency to improve the denoising performance of the BS signal. In addition, we calculate the time-domain features, frequency-domain features, and time–frequency-domain features of the BS signal obtained from the measurement, respectively, for downscaling the BS signal and preserving the original signal features.

### 3.1. Bowel Sound Signal Processing Method Based on Iterative Kurtosis Detector Algorithm and Kalman Filter

#### 3.1.1. Detector Algorithm Based on Iterative Kurtosis

Kim [31] proposed a modified iterative kurtosis-based detector (mIKD) algorithm, which is used to separate the bowel sound segments according to the measurement of kurtosis recorded along the sliding window.

The signal collected by the sensor is mainly composed of two parts: regular background noise, including lung sound and heartbeat sound generated by normal physiology. Theoretically, the kurtosis of these sounds is zero. The other part is composed of irregular bowel sounds, which represents the burst sound generated during intestinal peristalsis. The kurtosis of this kind of sound is positive. Therefore, the deviation between the kurtosis of the sound and zero value can be used to identify the existence of non-stationary transient bowel sounds.

BS and BGS, respectively, represent the inherent bowel sound and background signal. For the convenience of signal processing, the recorded sound (x=x(t):t=1,2,...,N) can be expressed in the form of (1).

(1) x=BS+BGS

**Figure 4:**
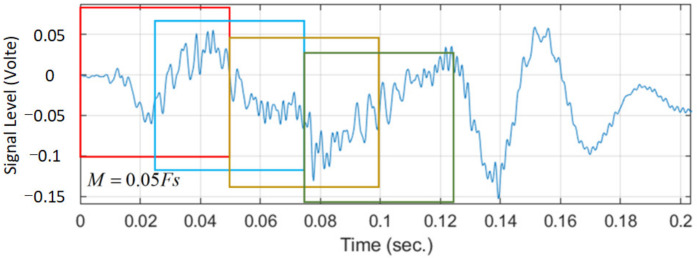
Sliding window.

The size of the sliding window in Figure 4 is defined as M = 0.05 × Fs, where Fs is the sampling frequency. In this paper, the length of each data record is 60 s, the size of the sliding window is set to 0.05, and the sliding speed is set to move 0.025 per second. Each data record is decomposed into 1200 segments during pre-processing by the sliding window. The size M of the sliding window will affect the reliability of kurtosis detection to recognize bowel sound signals. In general, the value of M is empirically determined.

**Figure 5:**
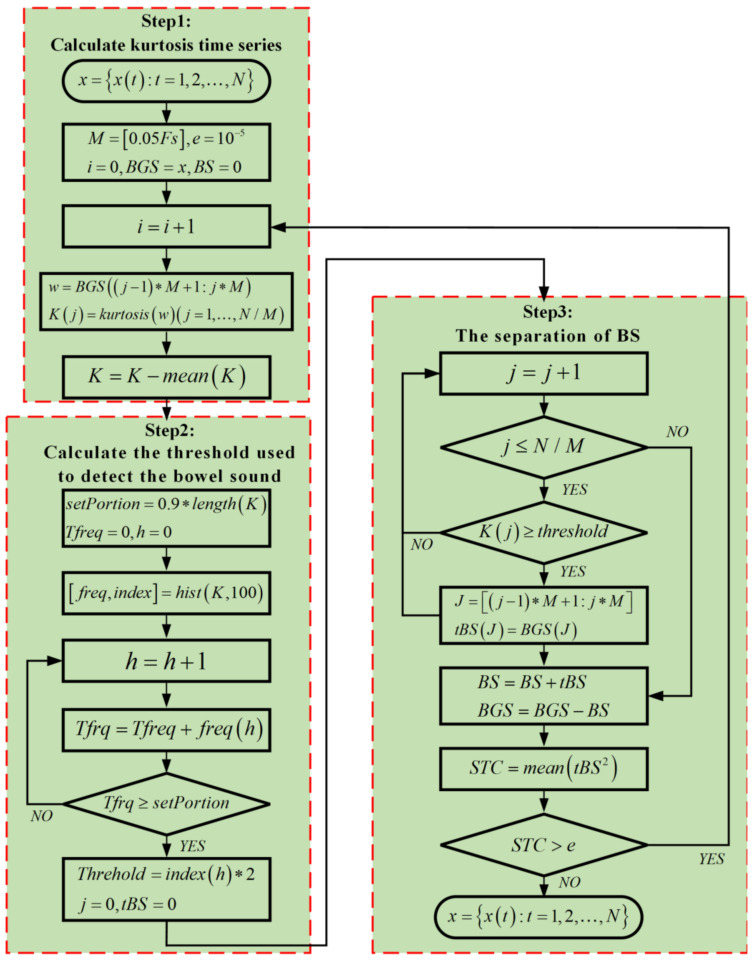
Flow chart of mIKD algorithm.

Figure 5 shows a flow chart of the algorithm. The mIKD algorithm is divided into three steps for iterative calculation, and BS in BGS are extracted according to the kurtosis threshold.

In Step 1, the kurtosis time series $K=K_{j}$ is calculated by the estimated average value $\hat{m}$ and estimated standard deviation $\hat{\sigma }$ of BGS in the j-th sliding window:

(2) $$K_{j}=\frac{\sum_{k=j}^{j+M-1}{\left[BGS\left(k\right)-\hat{m_{j}}\right]}^{4}}{\left(M-1\right){\hat{\sigma }}^{4}}-3$$

In Step 2, the distribution interval of the kurtosis time series K is calculated. The value of K is successively accumulated and counted according to each adjacent distribution interval, until the value of the current accumulated count (Tfreq) exceeds 90% of the total value of the kurtosis time series. Finally, the threshold is set to twice the range of the last interval.

In Step 3, the bowel sound is extracted from the BGS. The portion of the signal that exceeds the threshold is designated as a BS segment, while the rest is considered BGS. Repeated several times until the average power of the sound part is less than the pre-selected threshold, the algorithm iteration process is terminated. The final BS is the sum of all the BS parts in all the previous iterations, and the remaining signal is considered as the BGS signal.

Therefore, the BS signal, which is screened out in the j-th sliding window according to the threshold, can be expressed as:

(3) $$\begin{array}{cc} \; & BS((j-1)*M+1:j*M)=\\ \; & \left\{\begin{array}{cc} BGS((j-1)*M+1:j*M) & ,\;\mathrm{if}\;K(j)\geq Threshold\\ 0 & ,\;\mathrm{otherwise}. \end{array}\right. \end{array}$$

#### 3.1.2. Kalman Filter

Although the aforementioned mIKD algorithm has carried out segmenting screening of bowel sounds, the screened signals are still the aliasing state of signal truth value and noise. Therefore, the Kalman filter is used in this section to separate signal truth value and noise. The Kalman filter is essentially the process of estimating the real value based on the data of observation value and estimated value.

p(t) is continuous and second-order differentiable, which represents the “basic component” of the time series and actually reflects the trend of the time series, while b(t) is noise, which leads to randomness of the series and has no significance for the trend of time series.

The BS signals screened in Section 3.1.1 can be expressed as:

(4) BS(t)=p(t)+b(t)

Therefore, The Kalman filter is used to obtain an estimate of the time series p(t), that is, to achieve the denoising of the acquisition time series BS(t). For p(t), after the second-order Taylor expansion, that is

(5) $$\begin{array}{c} p\left(t\right)=p\left(t_{0}\right)+\frac{\partial p(t)}{\partial t}{|}_{t=t_{0}}\left(t-t_{0}\right)+\frac{1}{2}\frac{\partial^{2}p\left(t\right)}{\partial t^{2}}{|}_{t=t_{0}}{\left(t-t_{0}\right)}^{2}\\ +\sum_{n=3}^{\infty }\frac{1}{n!}\frac{\partial^{n}p\left(t\right)}{\partial t^{n}}{|}_{t=t_{0}}{\left(t-t_{0}\right)}^{n} \end{array}$$

Ignoring the higher-order small quantities and discretize time, that is

(6) $$p\left(t\right)\approx p\left(t_{0}\right)+v\left(t_{0}\right)h+\frac{1}{2}a\left(t_{0}\right)h^{2}$$

where $v\left(t_{0}\right)=\frac{\partial p(t)}{\partial t}{|}_{t=t_{0}}$ is the first derivative of the signal value, representing the speed value of the signal change, $a\left(t_{0}\right)=\frac{\partial^{2}p\left(t\right)}{\partial t^{2}}{|}_{t=t_{0}}=\frac{\partial v(t)}{\partial t}{|}_{t=t_{0}}$ is the second derivative of the signal value, and h is the time step.

$s\left(t\right)=\left[\begin{array}{c} p(t)\\ v(t)\\ a(t) \end{array}\right]$, $A=\left[\begin{array}{ccc} 1 & h & \frac{1}{2}h^{2}\\ 0 & 1 & h\\ 0 & 0 & 1 \end{array}\right]$, and $H=\left[\begin{array}{ccc} 1 & 0 & 0 \end{array}\right]$; w∼N(0,Q) represents the measured Gaussian noise, whose mean value is 0, *Q* is the covariance matrix of *w*, and ε∼N(0,R) represents the measured Gaussian noise, whose mean value is 0 and *R* is the covariance matrix of ε.

The prediction and measurement equations of the Kalman filter can be written as follows:

(7) s(t)=As(t−1)+Bu(t−1)+w

(8) BS(t)=Hs(t)+ε

It should be noted that Bu(t−1) represents the influence of the environment on the time series. This item can be omitted, as the trend of time series cannot be affected in general.

According to the method of Kalman filtering, the trend of time series p(t) after denoising can be obtained according to the measured value BS(t). Since the measured value and the estimated value have almost exactly the same probability distribution, in order to achieve a better denoising effect, the measured value of the previous (1∼N) time nodes can be randomly used as the measured value of the current time node under the assumption that the measured object does not change significantly. In principle, the larger the value of *N* is, the better the filtering effect will be, but it will also lead to more serious lag in filtering results.

**Figure 6:**
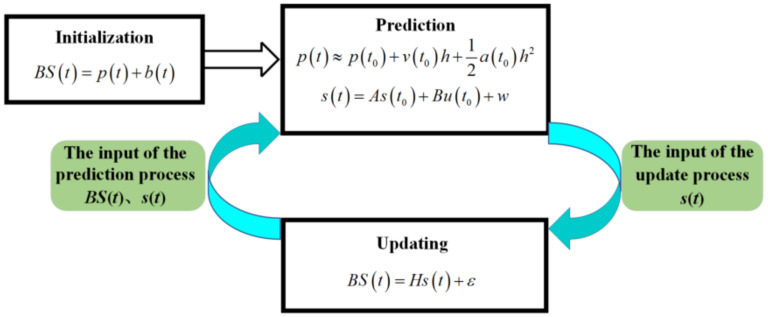
Prediction and updating process of BS signal by the Kalman filter.

As shown in Figure 6, the Kalman filter process of a BS signal is as follows: First initialize series BS(t). Then, the actual value p(t) is predicted by (5) and (6), and s(t) is used as the input of the update process. Then, the original series is updated according to (7), and the output p(t) is updated. s(t) and BS(t) are the input of the prediction process; the next round of prediction is repeated.

### 3.2. Feature Extraction in the Time Domain, Frequency Domain, and Time–Frequency Domain

After the previous data processing algorithm, directly using the original time series for neural network training is not only inefficient but will also affect the accuracy and reliability of the network fitting results due to its characteristics of frequent short-term fluctuations, noise interference, and unsteady state. Therefore, on the basis of the original data, three characteristic domains of time domain, frequency domain, and time–frequency domain are analyzed to ensure the dimensionality reduction in the original data and maintain all the characteristics of the original time series.

#### 3.2.1. Feature Extraction Based on Time Domain

BSs have the characteristics of long interval time, short duration, and unclear signal law in time–domain space. Compared with regular BGS, BS has an obviously unsteady state.

BS has large amplitude relative to BGS and is easy to distinguish. Therefore, the time-domain features such as mean and variance, extreme point, square root amplitude, absolute mean, skewness, and kurtosis are selected to screen and classify BS.

n is used to represent the size of a statistical length of time, i is used to represent the *i*-th row of data, and the time-domain features can be expressed as:

(1)Meanandvarianceofbowelsound

As a statistic reflecting the overall change characteristics of time series, the mean and variance of bowel sound signal can well reflect the change information of bowel sound time series, which are, respectively, defined as:

(9) $$TF_{1}=\frac{1}{n}\sum_{i=1}^{n}x(i)$$

(10) $$TF_{2}=\frac{1}{n}\sum_{i=1}^{n}{\left(x\left(i\right)-\bar{x}\right)}^{2}$$

(2)Extremepointofbowelsoundsignal

The extremum of bowel sound signal is an important feature of the segmental sub-sequence of bowel sound signal, which reflects the variation of bowel sound signal sequence in segmented interval to some extent. For a period of time-series $x_{i}\;\left(i=1,2,...k\right)$, the local maximum $maxS_{i}$ and minimum $minS_{i}$ of bowel sound signal are extracted, respectively:

(11) $$TF_{3}=max\left\{x(1),x(2),...,x(N)\right\}$$

(12) $$TF_{4}=min\left\{x(1),x(2),...,x(N)\right\}$$

(3)Squarerootamplitudeofbowelsoundsignal

The amplitude of bowel sound signal shows the activity of bowel sound signal time series to a certain extent.

(13) $$TF_{5}=X_{r}={\left(\frac{1}{n}\sum_{n=1}^{N}\sqrt{|x(n)|}\right)}^{2}$$

(4)Absolutemeanvalueofbowelsoundsignal

Compared with the mean value, the absolute mean value of the intestinal sound signal can better reflect the actual situation of the predicted value error because the value is absolute, so there is no positive and negative offset.

(14) $$TF_{6}=\frac{1}{N}\sum_{n=1}^{N}|x(n)|$$

(5)Skewnessandkurtosisofbowelsoundsignal

The skewness of bowel sound signal can be used to measure the asymmetry of the probability distribution of random variable of bowel sound signal. The kurtosis of bowel sound signal can be used to measure the steepness of the probability distribution of bowel sound signal and reflect the peak degree of bowel sound signal waveform.

(15) $$TF_{7}=\frac{1}{N}\sum_{n=1}^{N}{\left(x(n)\right)}^{3}$$

(16) $$TF_{8}=\frac{1}{N}\sum_{n=1}^{N}{\left(x(n)\right)}^{4}$$

#### 3.2.2. Feature Extraction Based on Frequency Domain

Fourier transform is carried out for BS time series. BGS frequency is concentrated between 0–100 Hz, and signal amplitude is large and dense. In contrast, BSs have frequent short-term fluctuations and are scattered between 200–1000 Hz, so the signal amplitude is greatly affected by frequency.

BS has a large frequency and small signal amplitude relative to the BGS. Therefore, according to the frequency characteristics of the signal, DC component, amplitude, and power spectral density are selected to extract the BS. Details are as follows:

(1)DCcomponentofbowelsoundsignal

The DC component represents the low-frequency characteristics of bowel sound signal.

(17) $$TF_{9}=\lim_{T\to \infty }\frac{1}{2T}\int_{-T}^{T}f\left(t\right)dt$$

(2)Amplitudeofbowelsoundsignal

Amplitude is the absolute value of bowel sound signal after Fourier transform, through which the energy value of intestinal sound signal at a specific frequency can be obtained. Where $F\left[\cdot \right]$ is the Fourier transform of the signal.

(18) $$TF_{10}=|F\left[x(t)\right]|$$

(3)Powerspectraldensityofbowelsoundsignal

The power spectral density of bowel sound signal describes the energy distribution of bowel sound signal data in the frequency domain.

(19) $$TF_{11}=\int_{-\infty }^{\infty }|F\left[x(t)\right]|$$

#### 3.2.3. Feature Extraction of Time Series Based on Multilayer Haar Wavelet Transform

The statistical characteristics in time domain or frequency domain describe the signal state in the whole statistical time, but the frequency composition information of vibration signal in a small range cannot be observed with the low time–frequency resolution. In addition to extracting characteristic parameters in the time domain and frequency domain, this section also takes the characteristic parameters in the time–frequency domain into consideration. Haar wavelet transform is used to extract the characteristics in the time domain and frequency domain, so as to comprehensively extract the characteristics of the signal.

The length of a bowel sound time series $BS=\left\{BS(t):t=1,2,...,N\right\}$ is n, and the number of layers of the Haar wavelet transform can be calculated according to the data length: since the complexity of the Haar wavelet transform is O(N), the $log_{2}\left(N\right)$-layer Haar wavelet transform can be performed on this bowel sound signal.

As shown in Table 1, in order to calculate the low-frequency signal of bowel sound signal in the *j* layer:

(20) $$A_{j}=a_{0,j},a_{1,j},...,a_{n-1,j}$$

and the high-frequency signal of bowel sound signal in *j* layer

(21) $$D_{j}=d_{0,j},d_{1,j},...,d_{n-1,j}$$

The low-frequency signals of bowel sound signal in the j−1 layer are divided into even parts:

(22) $$even\left(A_{j-1}\right)=a_{0,j-1},a_{2,j-1},...,a_{2n-2,j-1}$$

and odd parts:

(23) $$odd\left(A_{j-1}\right)=a_{1,j-1},a_{3,j-1},...,a_{2n-1,j-1}$$

Then, the low-frequency part $A_{j}$ of bowel sound signal in the *j* layer is:

(24) $$A_{j}=\frac{1}{\sqrt{2}}\left(even\left(A_{j-1}\right)+odd\left(A_{j-1}\right)\right)$$

the high-frequency part $D_{j}$ of bowel sound signal in the *j* layer is:

(25) $$D_{j}=\frac{1}{\sqrt{2}}\left(even\left(A_{j-1}\right)-odd\left(A_{j-1}\right)\right)$$

Thus, the features of the k-layer $k(k\in 1,2,...,log_{2}\left(N\right))$ wavelet transform of time-series $BS=\left\{BS(t):t=1,2,...,N\right\}$ are:

(26) $$W_{k}=\left[A_{k},D_{k},...,D_{2},D_{1}\right]$$

## 4. Prediction Model of Defecation Based on BP Neural Network

### 4.1. Prediction Model

**Figure 7:**
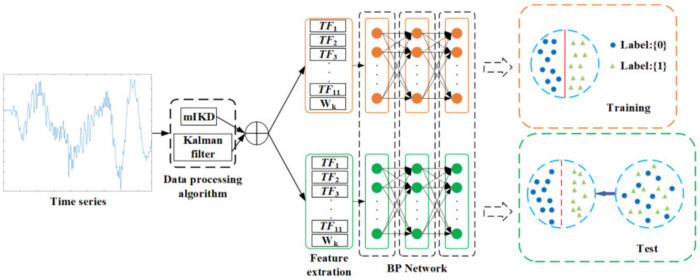
Prediction model of defecation based on BP neural network and bowel sound characteristics.

The prediction model of defecation based on BP neural network is shown in Figure 7. After the data processing of a bowel sound time series by mIKD algorithm and Kalman filter, appropriate time-domain, frequency-domain, and time–frequency-domain features $\left(TF_{1}∼TF_{11},W_{k}\right)$ are extracted as the input of neural network training process and test process, respectively, so as to generate the best weight to combine the global prediction.

In the training process (orange part of Figure 7), firstly, the appropriate features are selected for the input layer. Those containing defecation signals are denoted as class “1”, representing defecation; those without defecation signals are denoted as class “0”, representing no defecation. Training and weight updating of the classification network are carried out by artificially setting classification boundaries.

According to the pre-set data categories and labels, the prediction is evaluated and optimized by using the loss function, and the errors between neuron output of each layer and actual training labels are calculated layer by layer. The gradient descent method is adopted to minimize training errors. At the same time, parameters and weights of each layer of the network are saved. In the BP neural network model, there are three layers of structure: input layer, hidden layer, and output layer. For the i-th neuron, $x_{1},x_{2},...,x_{t}$ are the input of the neuron; the input is the independent variable of the key influence on the system model, and $w_{1},w_{2},...,w_{t}$ is the connection weight to adjust the weight ratio of each input.

The net input of $Net_{in}$ neurons can be obtained by selecting the most convenient linear weighted sum:

(27) $$Net_{in}=\sum_{n=1}^{n}\omega_{i}*x_{i}$$

In order to transform the features extracted into a group of weights, $\theta_{i}$ represents the threshold value of the neuron, and $Net_{in}$ is compared with $\theta_{j}$ and then processed by activation function

(28) $$y_{j}=f\left(Net_{in}-\theta_{j}\right)$$

where *f* is the activation function.

In a certain training example $\left(x_{k},y_{k}\right)$, the error of the prediction result can be expressed by the least square method:

(29) $$E_{k}=\frac{1}{2}\sum_{j=1}^{l}{\left(y_{j}-y_{k}\right)}^{2}$$

The numerical solution is found by iterative gradient descent to minimize the error between the actual output result and the expected output result. The search for the minimum value of the function may go through multiple iterations, and in each iteration, the weight between the nodes of each layer will be constantly updated iteratively.

(30) $$w\left(t+1\right)=w\left(t\right)-\eta \frac{\partial E_{k}}{\partial w}+\alpha \left[w(t+1)-w(t)\right]$$

where η represents the learning rate, $\frac{\partial E_{k}}{\partial w}$ is used to control the step size of iterative learning, and $\alpha \left[w(t+1)-w(t)\right]$ is the smoothing term.

**Figure 8:**
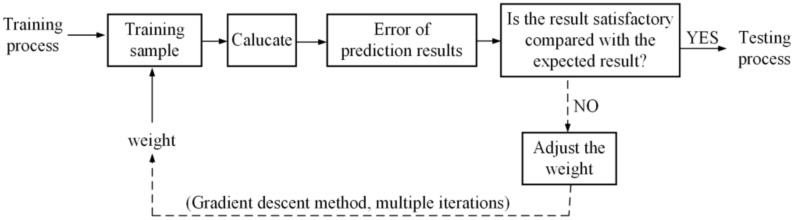
Weight updating process.

As shown in Figure 8, each iteration will produce a weight update and then forward propagate the updated weight with the training sample. The reverse propagation is repeatedly carried out to continue the iteration until obtaining a satisfactory result.

In the prediction process (the green part of Figure 7), the parameters and weights learned in the network are used to classify the unlabeled data into class “1” and class “0” to generate the prediction accuracy of upper and lower boundaries and true values. An input layer is designed to input the prediction data without labels into the BP neural network, which has learned the optimal weight, and generate all the prediction results of the network for this section of test data, comparing the network prediction results with the actual convenience labels and calculating the prediction accuracy of the convenience classification model based on the BP neural network.

### 4.2. Evaluating Models

In order to verify that the proposed method can achieve better performance than other classification methods, this study uses three basic metrics for different algorithm performance validation, respectively. In the binary classification model, the three metrics can be expressed as:

(31) $$Accuracy=\frac{TP+TN}{TP+TN+FP+FN}$$

(32) $$Sensitivity=\frac{TP}{TP+FN}$$

(33) $$Specificity=\frac{TN}{TP+FP}$$

True positive (TP), true negative (TN), false positive (FP), and false negative (FN) (are shown in Table 2) and were used as the main measures of performance. The values of accuracy, sensitivity, and specificity can evaluate the accuracy and uniqueness of the model in predicting the respective positive and negative sample categories.

## 5. Experiment Based on Bowel Sounds

### 5.1. Bowel Sound Signal Collection Experiment Reappearance

**Figure 9:**
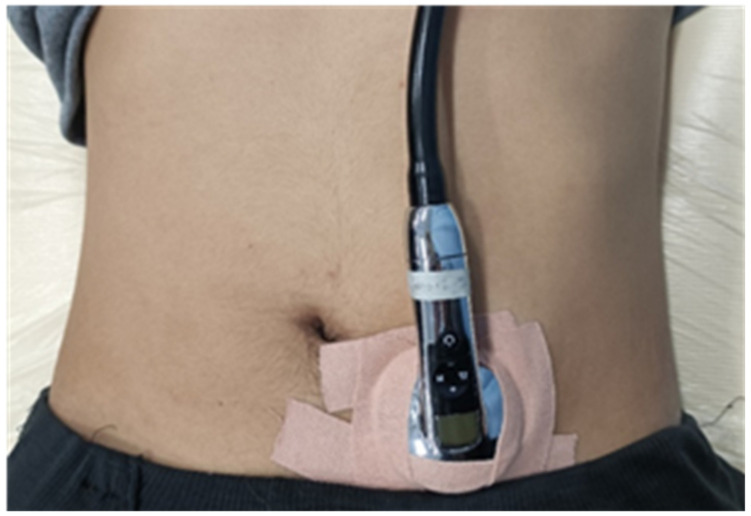
Bowel sound signal measured with an electronic stethoscope.

Using the bowel sound information obtained by 3M Littmann 3200 electronic stethoscope to extract the bowel sounds of the subjects as shown in Figure 9.

The 3200 electronic stethoscope incorporates ambient noise reduction technology, frictional noise damping materials, electronic amplifying (traditional bell/membrane or extended mode), Bluetooth data transmission, and accompanying Littmann StethAssist software to receive sound from inside the patient’s body. The bowel sounds of children or adults is auscultated and recorded in high quality, and the sound sampling frequency is 4000 Hz.

The physiological signals of the bowel sounds of volunteers are collected at three time periods one hour after breakfast, lunch, and dinner. According to the state of the subjects, they are divided into defecation intention and non-defecation intention. The data, 15 min before defecation, are marked as defecation intention, and the data in other time periods are non-defecation intention. When collecting data, volunteers are required to lie in bed and keep quiet to ensure stable breathing.

We collected 232 appropriate intestinal sound signal datum for experimental analysis from 6 volunteers, including patients from Beijing Bo-ai Hospital. Each single datum includes a 240,000-dimension bowel sound signal time series, and the volunteers range in age from 23 to 88 years old.

**Figure 10:**
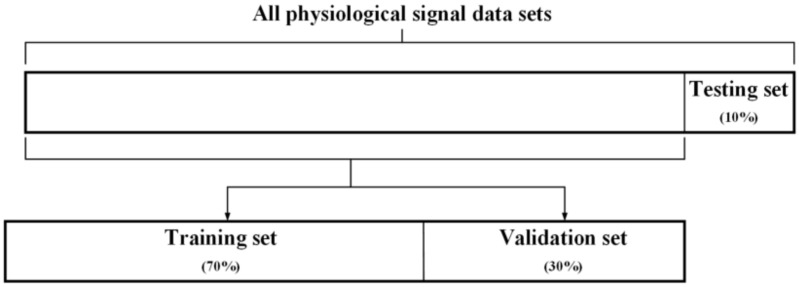
Data set of bowel sound signals assigned for training and testing.

In data allocation, as shown in Figure 10, the ratio of 7:3 is used to randomly allocate training sets and test sets. In 9/10 of all physiological signal data sets, 7/10 of the data are used as the training data set for network training, and the rest of the data are used as the test data set to test the accuracy of the network. Another 3/10 of the physiological signal dataset are used to evaluate the performance of our proposed system.

### 5.2. Signal Processing Results

#### 5.2.1. Kalman Filter Denoising

**Figure 11:**
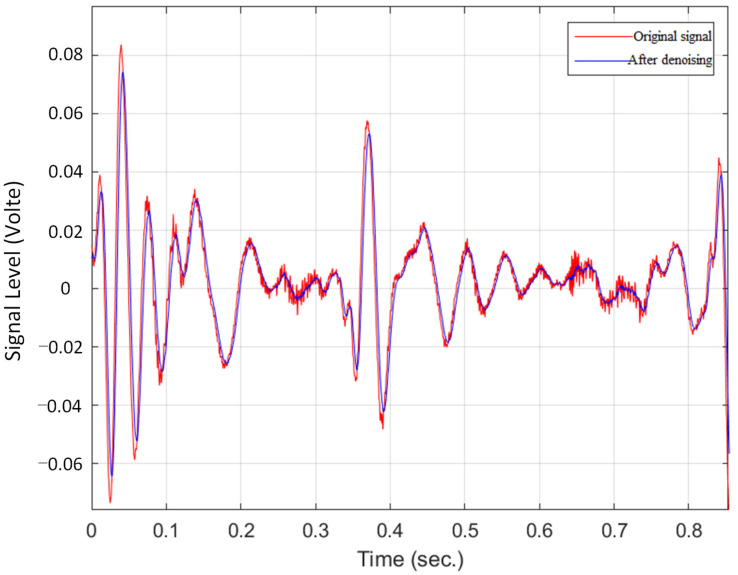
Original and denoising bowel sound signal.

Figure 11 shows the original data and the signal diagram after Kalman filter denoising.

The result shows that the noise in the environment is excellently removed after Kalman filtering.

#### 5.2.2. mIKD Processing

**Figure 12:**
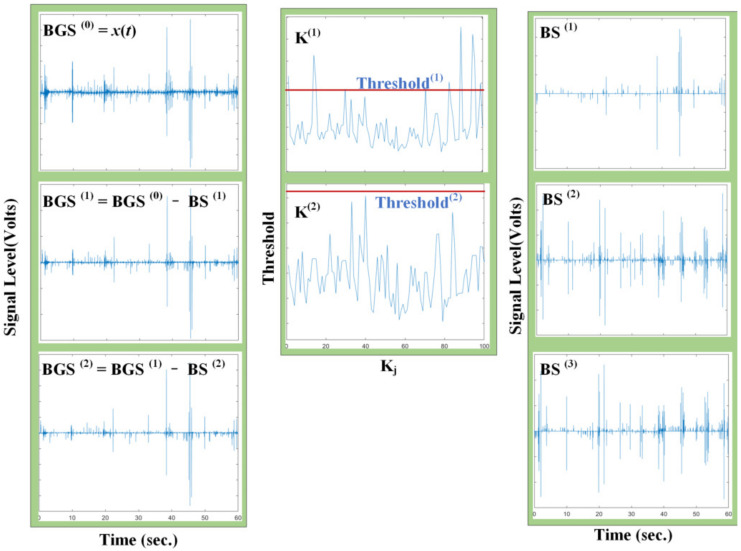
Signal processed by mIKD algorithm.

Figure 12 shows an example of the mIKD algorithm, showing the intermediate and final results obtained when applying mIKD to a bowel sound signal with a time length of 60 s. The result shows that the mIKD algorithm can separate the BS signal from the BGS signal easily.

The first column of the figure shows the estimated BGS at iteration i, $BGS^{\left(i\right)}$, and the last subgraph of the first column is estimated as the BGS residual signal, $BGS=BGS^{\left(2\right)}$. The second column describes kurtosis time-series K and the estimated threshold $Threshold^{\left(i\right)}$. According to the determination method of (3), when kurtosis time-series K exceeds the estimated threshold, $Threshold^{\left(i\right)}$, the BGS series corresponding to the kurtosis time series is extracted. The iteration ends when the kurtosis time-series K are all lower than the estimated threshold, $Threshold^{\left(i\right)}$. The final subgraph of the third column gives the final BS estimate, which is the sum of all previous estimates of $BS^{\left(i\right)}$.

### 5.3. Accuracy Verification of BP Neural Network

In order to verify whether the training data and test data are abnormal, multiple rounds of testing are needed to determine whether the predicted results of one round are random. In this paper, 20 rounds of repeated training and testing were conducted for each data set, and training data sets were randomly selected from the data set for each round. Among them, 20 rounds are based on experience. A small value will affect the judgment of whether the prediction result of this round is random error, and a large value will increase the calculation amount.

In one round of training, standard back propagation of batch 10 was used. Hidden nodes, learning rate, and iteration times were set to 200, 0.01, and 50,000, respectively. Loss index was used to evaluate the fitting degree of this model on the training set during training. In order to avoid overfitting during training, we defined that the training process was judged to be a convergence when the the difference of loss value between the two iterations was less than 0.01. In the testing process of this round, the final feature vectors are imported into the trained neural network, the training weights are used for testing, and the prediction results are calculated.

**Figure 13:**
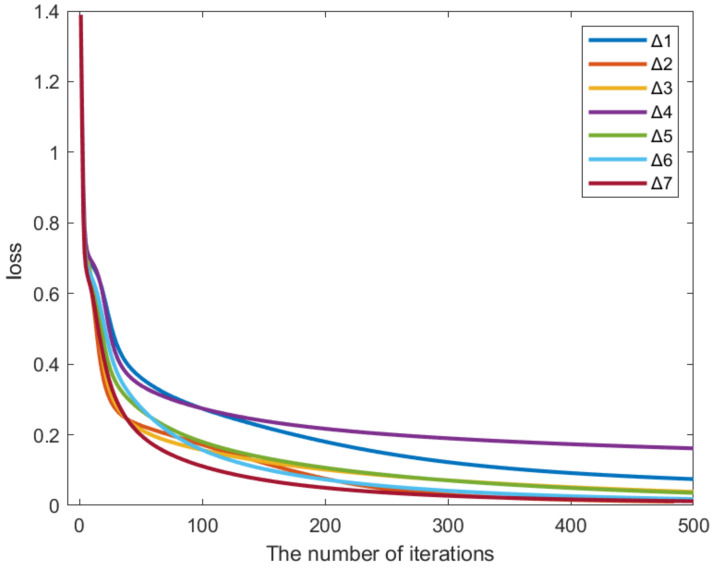
Loss curve of the network training process.

In order to test the accuracy and generalization of training data and test data, the bowel sound signal data of each volunteer were trained and tested separately, and finally, all bowel sound signals were cross-trained and tested. The test results are shown in Table 3, where represents the average results of individual training and testing of separate volunteers, and represents the average results of the cross-training and testing of all bowel sound signals of all volunteers. The final loss value calculated by is shown in Figure 13.

The prediction accuracy of our proposed model shows good performance, reaching 95.65%, 88.46%, 92.73%, 86.15%, 86.05%, and 87.93%, respectively, in six real data sets, and the average prediction accuracy based on mixed data sets is 88.71%. This indicates that our method can be applied to subjects of all ages, has universal applicability, and can achieve high accuracy.

According to our pre-defined method for determining the convergence of the curve, when the curve reaches 500 iterations, the loss value of the curve plateaus, and the error of the loss value in two iterations is less than 0.01, at which point the iteration stops. At the same time, the prediction accuracy is calculated based on test datasets. In all seven datasets, the loss values during the model prediction can reach a state of convergence, indicating the stability of our proposed model.

Table 4 shows the results of in 20 rounds of testing. The results show that the highest prediction accuracy was 94.29% in 20 rounds of tests, and 5.71% of intestinal sounds were misclassified. The prediction accuracy was the lowest in the first, seventeenth, and eighteenth rounds, with 85.71% of bowel sounds correctly classified and 14.29% incorrectly classified. The average accuracy of the 20-round test is 88.71%, which verifies the accuracy of the network prediction and indicates that the BP neural network algorithm based on feature extraction in time domain, frequency domain and time–frequency domain can better realize the defecation prediction.

### 5.4. Performance Evaluation of BP Neural Network

Based on bowel sound signal, this paper adopts multiple feature spaces to represent the waveform data of bowel sound signals, such as the original feature space, including time, frequency, and time-frequency, to ensure the integrity of the original information. To evaluate the performance of our calibration model, we compare our method with the methods in [16,17,18] in terms of prediction accuracy.

Firstly, Naive Bayes (NB), Logistic regression algorithm (Logistic), and Decision tree algorithm (C4.5) are used to construct a bowel sound signal-based defecation prediction model and then a training data set based on the filtering and feature extraction methods with the same label. These signals are denoted as class “1”, representing defecation; the other signals are denoted as class “0”, representing no defecation. At last, the analysis compared the prediction accuracy of each method using the bowel sounds from the same electronic stethoscope.

Table 5 offers a detailed comparative study of the three metrics (accuracy, sensitivity, and specificity) of four algorithms explicitly. The results indicate, for single models, that the BP classifier has the highest values in accuracy, sensitivity, and specificity among all models, respectively, reaching 91.4%, 92.1%, and 90.6%. At the same time, the C4.5 and NB methods exhibited ineffectual outcomes with the least values of accuracy. The logistic classifier approaches reached somewhat higher values of accuracy compared with the other two.

**Figure 14:**
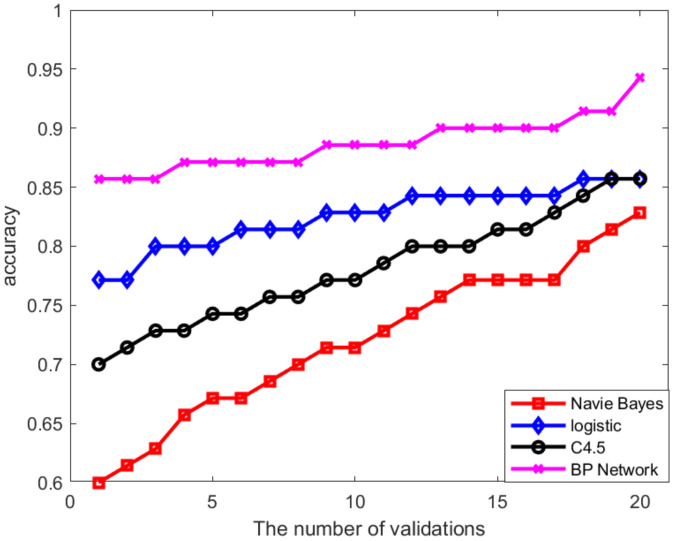
Average prediction accuracy of each model.

Figure 14 show the prediction accuracy of each model based on the Δ7 data sets. The results demonstrate that the NB and C4.5 algorithms illustrate relatively poor prediction performance, with an average prediction accuracy of 68.43% and 75%, respectively. The logistic algorithm model obtained slightly increased values of accuracy, with an average prediction accuracy of 82.5%. Afterward, the BP neural network demonstrated the best performance of all classifiers, with an average prediction accuracy of 88.71%, and all three metrics (accuracy, sensitivity, and specificity) are better than the other classifiers (NB, C4.5, and logistic).

Table 6 shows the prediction accuracy of each model based on different domain features. The results show that the training based on multidomain features was the best, with an average prediction accuracy of 80.64%. Furthermore, each of the individual domain-based features (time-domain features, frequency-domain features, and time–frequency-domain features) was slightly worse, with average prediction accuracies of 68.35%, 64.38%, and 73.80%, respectively. Table 7 shows the prediction accuracy of each model based on different domain feature combinations. The results show that the features of any domain are meaningful and that removing features from any of the domains will make the prediction less accurate. Furthermore, it can be seen from Table 6 and Table 7 that the BP neural network has better prediction performance than the other classifiers

Our study shows that NB, logistic, C4.5, and the BP neural network have similar classification performances based on the same feature space and perform well in the classification of bowel sound signals based on time-domain, frequency-domain, and time–frequency-domain features. However, each learning mode has its own disadvantages:

**1.** The NB algorithm has a small error classification rate, but when the number of attributes is large or the correlation between attributes is large, the classification effect of the NB model is relatively poor.

**2.** The logistic algorithm is suitable for the automatic classification of class domains with relatively large sample size, but the algorithm has a large amount of calculation, and the output of the algorithm is not strong enough to be explained. The classification results are more unstable in the training of small samples.

**3.** C4.5 uses gain rate to select attributes, resulting in classification rules that are easy to understand and have high accuracy but requires sequential scanning and sorting of data sets many times, resulting in low efficiency of the algorithm.

**4.** The BP neural network has high classification accuracy and strong distributed parallel processing ability, with strong robustness and fault tolerance for data sets containing a large number of noise data.

Compared with the other three classification models, the prediction result of the BP neural network is improved by 6.21% compared with the best classification result of the other three classification methods and 15.43% compared with the worst classification result of the other three classification methods. The effect is more accurate and the performance is better.

## 6. Discussion

The main weakness of this work is the lack of repeatability, and this results from the minimal information about the subjects of the study and demographics data, as well as the clinical conditions of patients. In fact, if our proposed system is able to accurately classify the presence or absence of bowel movements in elderly people with disabilities, then once our proposed model is sufficiently trained, the system could identify unknown bowel sound signals to some extent. Relying on a transfer learning approach, we also intend to extend this approach to other gastrointestinal disorders such as acute bowel obstruction, renal failure, and acute appendicitis.

Although machine learning provides powerful algorithms for the classification of bowel sounds and the prediction of bowel movements, single machine learning algorithms often tend to fall into local optima, the model prediction accuracy is still not very high, and it is difficult to explain its learning process. Therefore, the use of ensemble learning combines the outputs of multiple techniques and machine learning models with the aim of improving prediction accuracy through a weighted average approach to obtain improved prediction estimates and ranges [32]. As part of our future research, we intend to improve the performance and reliability of our proposed system by applying ensemble learning methods in the next step of our work and obtaining more data on bowel sound signals.

## 7. Conclusions

Early warning of incontinence can help provide timely treatment and improve the quality of life of disabled older persons. As the main tool for monitoring bowel diseases, bowel sound provides a solution for bowel early warning in patients with incontinence. In this work, we use the BP neural network to determine the feasibility of the bowel intention assessment model based on the characteristics of bowel sound signals. We use a detector algorithm based on iterative kurtosis and a signal processing method based on the Kalman filter to process the signal and extract the signal features from the time domain, frequency domain, and time–frequency domain. The BP neural network is selected to carry out training classification networks on the extracted features and build the training classification method.

We evaluated the effectiveness of our proposed method using data sets collected by an electronic stethoscope-based intestinal rumble signal acquisition system. The proposed method converges to a stable state after 500 iterations, and the average classification accuracy of the model test results is 88.71%. This indicates that our model can recognize the classification of defecation based on bowel sound signals even in the presence of noise. Compared with the Naive Bayes, logistic, and C4.5 classification models, the accuracy of the prediction results is better than that of the other classification models, which verifies the validity and practicability of the proposed method. This shows that the overall performance of our proposed system is good enough to help doctors make diagnoses.

The main highlights of the proposed defecation prediction model based on the BP neural network and bowel sound signal features are as follows:

1. The feature learning process is simple and does not require much calculation, nor do we need to manually develop a set of optimal features to feed into the BP neural network.

2. The performance of our proposed method will improve with the amount of data. When more data is used for training, our system will achieve better performance.

On the whole, the prediction and validation results of the BP neural network are higher than those of the other three classification networks, and the model performance is relatively good. This model can evaluate the intention of unknown bowel sounds to a certain extent.

## Figures and Tables

**Table 1:** Multilayer Harr wavelet transform.

Layers	Parameters				
0	$A_{0}$				
1	$A_{1}$				$D_{1}$
2	$A_{2}$			$D_{2}$	$D_{1}$
…	*…*			$D_{2}$	$D_{1}$
*j*	$A_{j}$	$D_{j}$	*…*	$D_{2}$	$D_{1}$
…	*…*			$D_{2}$	$D_{1}$
$log_{2}\left(N\right)$	$A_{k}$	$D_{k}$	*…*	$D_{2}$	$D_{1}$

**Table 2:** Confusion matrix of classification predictions.

	Predicted: 1	Predicted: 0
Actual: 1	TP	FP
Actual: 0	FN	TN

**Table 3:** Average accuracy of calculated Δ1∼Δ7.

Cases	Average Accuracy
Δ1	*95.65%*
Δ2	*88.46%*
Δ3	*92.73%*
Δ4	*86.15%*
Δ5	*86.05%*
Δ6	*87.93%*
Δ7	*88.71%*

**Table 4:** Prediction results of 20 rounds of Δ7.

Round	1	2	3	4	5
Accuracy	85.71%	91.43%	88.57%	88.57%	87.14%
Round	6	7	8	9	10
Accuracy	90.00%	87.14%	90.00%	90.00%	94.29%
Round	11	12	13	14	15
Accuracy	90.00%	90.00%	87.14%	87.14%	88.57%
Round	16	17	18	19	20
Accuracy	87.14%	85.71	85.71%	88.57%	91.43%

**Table 5:** Evaluation of each model.

Model	Accuracy	Sensitivity	Specificity
BP neural network	0.914286	0.921053	0.90625
C45	0.728571	0.631579	0.84375
NB	0.728571	0.631579	0.84375
Logistic	0.846154	0.848485	0.84375

**Table 6:** Classification accuracy of different classifiers with different domain features.

Different Classifiers	The Prediction Accuracy with Different Features (%)				Average Accuracy (%)
	Time Domain Features	Frequency Domain Features	Time-Frequency Domain Features	Multi-Domain Features	
BP nerual network	72.62%	73.39%	84.54%	88.71%	79.80%
C4.5	69.00%	66.14%	75.00%	78.07%	72.05%
NB	69.07%	56.86%	68.43%	73.28%	66.91%
Logistic	62.71%	61.14%	67.21%	82.50%	68.39%
Average accuracy (%)	68.35%	64.38%	73.80%	80.64%	—

**Table 7:** Classification accuracy of different classifiers with different domain feature combinations.

Different Classifiers	The Prediction Accuracy with Different Features Combinations (%)				Average Accuracy (%)
	Time and Frequency Domain Features	Frequency and Time–Frequency Domain Features	Time and Time–Frequency Domain Features	Multidomain Features	
BP nerual network	80.77%	88.15%	87.85%	88.71%	86.37%
C4.5	68.14%	74.50%	77.07%	78.07%	74.44%
NB	68.43%	69.00%	75.07%	73.28%	71.45%
Logistic	67.36%	82.36%	79.57%	82.50%	77.95%
Average accuracy (%)	71.18%	78.50%	78.89%	80.64%	—

## Data Availability

We created a dataset for this study. Since further research is in progress, we cannot publish the dataset right now.

## References

[1] Halland M., Talley N.J. (2012). Fecal incontinence: Mechanisms and management. Curr. Opin. Gastroenterol..

[2] Smith E.M., Shah A.A. (2017). Screening for Geriatric Syndromes. Clin. Geriatr. Med..

[3] Prather C.M. (2004). Physiologic variables that predict the outcome of treatment for fecal incontinence. Gastroenterology.

[4] Rudd T.N. (1959). Human problems in geriatrics, with special reference to fecal incontinence. J. Am. Geriatr. Soc..

[5] Tariq, Syed H. (2004). Geriatric fecal incontinence. Clin. Geriatr. Med..

[6] Yagüe T.M. (2006). Fecal and anal incontinence. Rev. Esp. Enfermedades Dig..

[7] Camilleri M., Bharucha A.E., Lorenzo C.D., Hasler W.L., Prather C.M., Rao S.S., Wald A. (2010). American Neurogastroenterology and Motility Society consensus statement on intraluminal measurement of gastrointestinal and colonic motility in clinical practice. Neurogastroenterol. Motil. Off. J. Eur. Gastrointest. Motil. Soc..

[8] Liem O., Van Den Berg M.M., Mousa H.M., Youssef N.N., Langseder A.L., Benninga M.A., Lorenzo C.D. (2010). Distention of the colon is associated with initiation of propagated contractions in children. Neurogastroenterol. Motil..

[9] Mantoo S., Meurette G., Podevin J., Lehur P.A. (2012). The magnetic anal sphincter: A new device in the management of severe fecal incontinence. Expert Rev. Med. Devices.

[10] Jiang E., Zan P., Zhang S., Liu J., Zhu X., Wang X. (2012). Mechanical Model of a Novel Executive Mechanism for Artificial Anal Sphincter System. International Computer Science Conference.

[11] Jelovsek J.E. (2015). Prediction Models for Postpartum Urinary and Fecal Incontinence in Primiparous Women: Erratum. Female Pelvic Med. Reconstr. Surg..

[12] Arnbjörnsson E., Bengmark S. (1983). Auscultation of Bowel Sounds in Patients with Suspected Acute Appendicitis—An Aid in the Diagnosis?. Eur. Surg. Res..

[13] Yoshino H., Abe Y., Yoshino T., Ohsato K. (1990). Clinical application of spectral analysis of bowel sounds in intestinal obstruction. Dis. Colon Rectum.

[14] Dalle D., Devroede G., Thibault R., Perrault J. (1975). Computer analysis of bowel sounds. Comput. Biol. Med..

[15] Kim K.S., Seo J.H., Song C.G. (2011). Non-invasive algorithm for bowel motility estimation using a back-propagation neural network model of bowel sounds. Biomed. Eng. Online.

[16] Fernández F., Isasi P. (2004). Evolutionary Design of Nearest Prototype Classifiers. J. Heuristics.

[17] AL-Ghamdi A.S.A., Ragab M., Sabir M.F.S., Elhassanein A., Gouda A.A. (2022). Optimized Artificial Neural Network Techniques to Improve Cybersecurity of Higher Education Institution. Comput. Mater. Contin..

[18] García-Pedrajas N., Fyfe C. (2008). Construction of classifier ensembles by means of artificial immune systems. J. Heuristics.

[19] DuBose J.J., Lissauer M., Maung A.A., Piper G.L., O’Callaghan T.A., Luo-Owen X., Inaba K., Okoye O., Shestopalov A., Fielder W.D. (2013). Pneumatosis Intestinalis Predictive Evaluation Study (PIPES): A multicenter epidemiologic study of the Eastern Association for the Surgery of Trauma. J. Trauma Acute Care Surg..

[20] Dimoulas C., Kalliris G., Papanikolaou G., Kalampakas A. (2007). Long-term signal detection, segmentation and summarization using wavelets and fractal dimension: A bioacoustics application in gastrointestinal-motility monitoring. Comput. Biol. Med..

[21] Dimoulas C., Kalliris G., Papanikolaou G., Petridis V., Kalampakas A. (2008). Bowel-sound pattern analysis using wavelets and neural networks with application to long-term, unsupervised, gastrointestinal motility monitoring. Expert Syst. Appl..

[22] Hadjileontiadis L.J. (2005). Wavelet-based enhancement of lung and bowel sounds using fractal dimension thresholding-part I: Methodology. IEEE Trans. Biomed. Eng..

[23] Hadjileontiadis L.J., Liatsos C.N., Mavrogiannis C.C., Rokkas T.A., Panas S.M. (2000). Enhancement of Bowel Sounds by Wavelet-Based Filtering. IEEE Trans. Biomed. Eng..

[24] Hadjileontiadis L.J., Rekanos I.T. (2003). Detection of explosive lung and bowel sounds by means of fractal dimension. Signal Process. Lett. IEEE.

[25] Ranta R., Louis-Dorr V., Heinrich C., Wolf D., Guillemin F. (2010). Digestive Activity Evaluation by Multichannel Abdominal Sounds Analysis. IEEE Trans. Biomed. Eng..

[26] Rekanos I., Hadjileontiadis L. (2006). An iterative kurtosis-based technique for the detection of nonstationary bioacoustic signals. Signal Process..

[27] Sandler R.H., Mansy H.A., Kimura R.A., Uhing M.R., Arango V. (1996). 107 computerized analysis of bowel sounds in normal and small bowel obstructed rats. J. Pediatr. Gastroenterol. Nutr..

[28] Radnitz C.L., Blanchard E.B. (1989). A 1- and 2-year follow-up study of bowel sound biofeedback as a treatment for irritable bowel syndrome. Biofeedback Self-Regul..

[29] Garner C.G., Ehrenreich H. (1989). Non-invasive topographic analysis of intestinal activity in man on the basis of acustic phenomena. Res. Exp. Med..

[30] Furness J.B., Callaghan B.P., Rivera L.R., Cho H.J. (2014). The Enteric Nervous System and Gastrointestinal Innervation: Integrated Local and Central Control. Oxyg. Transp. Tissue XXXIII.

[31] Kim K.S., Seo J.H., Sang H.R., Min H.K., Song C.G. (2011). Estimation algorithm of the bowel motility based on regression analysis of the jitter and shimmer of bowel sounds. Comput. Methods Programs Biomed..

[32] Dong X., Zhiwen Y.U., Cao W., Shi Y., Qianli M.A. (2019). A survey on ensemble learning. Front. Comput. Sci..

